# The sDOR.2-6y™ Is a Valid Measure of Nutrition Risk Independent of BMI-for-Age z-Score and Household Food Security Status in Preschool Aged-Children

**DOI:** 10.3390/nu16060767

**Published:** 2024-03-07

**Authors:** Elizabeth H. Ruder, Barbara Lohse

**Affiliations:** Wegmans School of Health and Nutrition, College of Health Science and Technology, Rochester Institute of Technology, Rochester, NY 14623, USA; balihst@rit.edu

**Keywords:** child eating behavior, parent feeding behavior, young children, food security, Satter division of responsibility in feeding, child eating behavior questionnaire, eating competence

## Abstract

Parents’ feeding practices are a function of child eating behaviors, health, and other factors. Adherence to the Satter Division of Responsibility in Feeding (sDOR) model has not been examined relating to child BMI, household food security, or child eating behavior. This study evaluates the adherence to sDOR in relation to child eating behavior, nutrition risk, BMI-for-age, dietary intake, and food security. Ninety-one parent–child (3 to <6 years) dyads completed a cross-sectional asymmetric survey in August–November 2019; *n* = 69 parents from the original sample completed additional and retrospective questions in June 2021. Main outcomes included sDOR adherence (sDOR.2-6y™), a Child Eating Behavior Questionnaire (CEBQ), nutrition risk (NutriSTEP^®^), the USDA 6-item screener, the Block Kids Food Screener, and eating competence (ecSI 2.0™). The children’s weight and height were investigator-measured. Associations were tested with Pearson’s r and Chi Square for continuous and categorical variables, independent sample *t*-test, one-way ANOVA, or Mann–Whitney U compared means. The dietary comparisons used Spearman’s rho correlation coefficient. sDOR adherence was associated with a lower nutrition risk (r = 0.26, *p* = 0.03) and showed convergent validation with child eating behavior for three child eating behavior (CEBQ) constructs. sDOR.2-6y™ was not related to the child BMI-for-age z-score (r = 0.11, *p* = 0.39, n = 69). NutriSTEP^®^ was associated with dietary quality and higher ecSI 2.0TM (r = 0.32, *p* = 0.008, n = 69). No associations between sDOR.2-6y™ and food security or dietary intake were noted.

## 1. Introduction

Feeding a young child is relational and multisystemic, involving physical, psychological, and developmental factors that are unique to the individual child, characteristics of the parent and family system, and environmental and contextual factors [1]. Disruption of any of these systems can put the child at risk of feeding disorders and related complications [2], and an estimated 25% to 50% of young children are reported to have feeding difficulties [3,4]. The Satter Division of Responsibility in Feeding (sDOR) model [5] establishes trusting feeding relationships for enjoyable mealtimes to prevent or treat concerns with feeding young children. The sDOR model has two tenets of equal importance: parent leadership in feeding and child autonomy in eating. The sDOR posits that caregivers provide leadership in terms of what foods are provided for the child to eat, the emotional and physical environment (where), and timing (when). Furthermore, caregivers provide autonomy support, meaning that they trust children’s competence to determine what, how much, and whether they eat from the food that is served. The sDOR differs from existing models that tend to focus on parents’ management of children’s eating [6,7,8,9,10,11,12,13] or maladaptation in children’s competence [14,15]. 

Adherence to the sDOR model is measured by the sDOR.2-6y™, a validated and reliable tool to assess parent/caregiver adherence to both sDOR tenets. The sDOR.2-6y™ was developed through a series of validation studies, including content validation [16]. Construct validation was achieved through in-home video capture to examine parents’ feeding behaviors, with 24 h recall dietary assessments to affirm meal timing compared with parent survey responses [17]. A 2021 publication [18] demonstrated that sDOR.2-6y™ scores were higher with lower restriction and lower pressure to eat (assessed by the Child Feeding Questionnaire) [11] and were associated with the feeding style (assessed by Caregivers Feeding Styles Questionnaire) [19]. These findings provided evidence of criterion validation, i.e., performance on one instrument compared to performance on another validated measure. In addition, convergent construct validation, i.e., whether constructs in the sDOR.2-6y™ and constructs in another validated instrument that theoretically should be related to each other are, was demonstrated by the ability of sDOR.2-6y™ to identify nutrition risk in young children as indicated by parents’ responses to the NutriSTEP survey^®^ [18]. 

Continued construct and criterion validation of sDOR.2-6y™ extends the application of the tool to clinical and community health settings. Criterion validation has been demonstrated between the sDOR.2-6y™ and the Child Feeding Questionnaire [11] and Caregivers Feeding Styles Questionnaire [19], but criterion validation has not been examined with the Child Eating Behavior Questionnaire (CEBQ) [20], which demonstrates cross-sectional relationships with measures of child adiposity [21]. Parental feeding behaviors/styles can enhance or undermine children’s ability to self-regulate and ultimately affect the child’s weight [22,23]. Despite the clinical opinion that sDOR in feeding adherence helps children recognize and respond to hunger, satiety, and appetite (i.e., self-regulate) [5], and evidence that parents’ feeding behaviors being in opposition to sDOR (i.e., controlling, restricting) are related to child obesogenic behaviors [24,25,26], an assessment of sDOR.2-6y™ with child weight status has not been performed. Moreover, existing validation studies of sDOR.2-6y™ were performed in predominately resource-constrained families, but without an assessment of food insecurity. This limits the applicability to a more general audience, since social factors related to income are known to influence parenting practices [27]. In addition, the presence of household food insecurity could be an important mediator of the feeding relationship in resource-constrained families [28,29]. To that end, the purpose of this study was to examine adherence to sDOR in relation to the child’s BMI-for-age z-score, household food security status, caregiver response to the CEBQ, and child’s dietary intake. An additional aim was to re-affirm the ability of sDOR.2-6y™ to identify nutrition risk in young children.

## 2. Materials and Methods

### 2.1. Study Design and Sample Recruitment

The target sample for recruitment was parents/caregivers of children ages 3 to <6 years. A convenience sample of six early childhood education facilities (i.e., childcare centers, Universal Pre-K Programs, and Universal Pre-K in a child care setting) located in the greater Rochester, NY, area agreed to allow for recruitment from the approximately 280 potential parent–child dyads meeting the child age requirements. The study design was a cross-sectional design of one parent/caregiver respondent sample, assessed twice with asymmetric survey completion. The reason for the dual survey administration was twofold: (1) Additional sDOR.2-6y™ questions about child feeding were validated, since the original survey administration occurred in 2019, and these additional questions were surveyed in the 2021 administration. (2) A clerical error in the 2019 survey design omitted one NutriSTEP^®^ question about screen time. This item was included for response retrospectively and currently in the 2021 sDOR.2-6y™ survey 2021. Data for the initial survey administration were collected in August–November 2019 and included select items of the sDOR.2-6y™. The complete 12-item survey was administered in June 2021 with the one NutriSTEP^®^ item on screen time. Children’s height and weight were collected concurrently with the first survey administration in 2019.

Recruitment materials that described the study and included a link to the online consent and online survey were distributed by the schools via email to parents/caregivers between August and November 2019. On accessing the link, potential participants confirmed the inclusion criteria. These included being able to read and speak English, being at least 18 years of age, receiving information on the study from their child’s child care/early Pre-K or Universal Pre-K center, having a child who was at least three (3) years old but not yet six (6) years old, being enrolled in a school where they learned about the study, and absence of any condition in the child which significantly affected eating or appetite (e.g., a severe food allergy or medical condition). Upon confirmation of these criteria, parents/caregivers provided consent to participate and continued to the written online survey set. As described previously, parents/caregivers in the 2021 survey administration responded to the NutriSTEP^®^ for the current time and thinking back retrospectively to fall 2019. The response for screen time in 2019, rather than in 2021, was used to calculate the NutriSTEP^®^ score, because screen time would be expected to be lower in younger age groups or unchanged, and only 3 parents noted that screen use was greater in 2019 than 2021 (all by 1 h), whereas 32 reported lower 2019 screen time, and 34 parents reported that screen time was unchanged.

Verbal assent was received from the child prior to measuring the child’s weight and height. Children’s weight and height were measured at the school during regular school hours. This study was approved, as no greater than minimal risk, through Expedited review, Category 7 exempt by Rochester Institute of Technology Human Subjects Research Office.

Parents/caregivers received a USD 30 retail gift card for initial participation, and the child received a small prize after getting weighed and measured. Parents/caregivers who completed the initial survey were re-contacted in June 2021 to complete additional questions. Individuals who completed that survey received an additional USD 20 retail gift card.

Planned sample size, based on the previous validation study [18], was 100. Since the sample completing both sDOR.2-6y™ and NutriSTEP^®^ did not reach 100, a post hoc power calculation was conducted to verify the power of the actual sample size. This was based on the sDOR.2-6y™ standard deviation in previous studies [16,18] (i.e., 3.3) and suggesting a clinically significant sDOR.2-6y™ score difference between risk groups of 2.5–3 points. Using the Altman nomogram [30], a sample size ≥55 would have a power of 0.8 to identify a significant sDOR.2-6y™ difference (<0.5) between groups at no nutrition risk or some risk, as indicated by NutriSTEP^®^ scores. This sample size was also adequate for detecting a significant association between nutrition risk groups and adherence or non-adherence to sDOR using a cutoff of ≥26 to indicate sDOR adherence, with an assumption of 5–10% and 40% of ≥26 and <26 sDOR.2-6y™ scores, respectively, being at nutrition risk.

### 2.2. Data Collection

The sDOR.2-6y™ was compared with validated and tested measures related to sDOR tenets. The online survey set employed validated and reliable questionnaires, as outlined and presented in the section below. All responses were provided by consenting parents/adult caregivers.

Dietary Intake: Children’s dietary intake was measured by the Block Kids Food Screener Ages 2–17 (BKFS) [31]. This brief dietary screening questionnaire is designed to assess children’s dietary intake during the previous week. The 41-item questionnaire focuses on intake of fruit and fruit juices, vegetables, potatoes (including French fries), whole grains, meat/poultry/fish, dairy, legumes, saturated fat, and added sugars (in sweetened cereals, soft drinks, and sweets). The questionnaire includes both a 6-point frequency of intake (ranging from “none last week” to “every day last week”) and 3-point quantity of intake that varies in unit by the food item (i.e., 1 glass, 1 egg, ½ a piece of fruit, etc.) [31]. Food group estimates are reported as average daily intake in cup equivalents (for fruit/fruit juice; vegetables excluding potatoes and legumes; potatoes, including French fries); ounce equivalents (for whole grains and meat, poultry, and fish); and teaspoons (for sugar/syrup added to foods/beverages during processing/preparation). The BKFS has been validated against the 24 h recall method [32] and was developed to address concerns about the time that is required to complete the longer Block Kids Food Frequency Questionnaire [33].

Satter Division of Responsibility: Adherence to the Satter Division of Responsibility in Feeding was measured with the 12-item sDOR.2-6y™. The tool can be used at no cost, but only upon approval of an application for use [34]. The Ellyn Satter Institute provided permission to use the sDOR.2-6y™ with scoring instructions. Each item includes 5 response options, assigned a value from 3 to 0. Six items are phrased so that a positive response indicates sDOR adherence; five items are interpreted as sDOR non-adherence; and one item denotes sDOR adherence as neither strong agreement nor disagreement, but rather a less frequent but apparent occurrence. Five sDOR.2-6y™ subscales are identified within the domains of parent leadership and child autonomy [18]. Parent leadership subscales include mealtime structure, what food is available to the child, and how food is available to the child; and child autonomy subscales include respect for child autonomy in eating and who controls what, when, or how much is eaten. Total sDOR.2-6y™ possible scores are 0 to 36 [17] and can be categorized into two domains: leadership and child autonomy, with possible scores of 0–21 and 0–15, respectively [18]. Parent leadership addresses mealtime structure, what food is available to the child, and how food is available to the child. Child autonomy considers parents’ respect for child autonomy in eating and who controls what, when, or how much is eaten. Scores ≥ 26 are recommended as indicating adherence to sDOR when sensitivity to detect nutrition risk is the concern [18].

Child Eating Behavior: Child eating behavior was assessed by the Children’s Eating Behavior Questionnaire (CEBQ), a 35-item survey to assess eating style in young children based on parent-reported child behavior [20]. Items are scored on a 5-point scale (Never, Rarely, Sometimes, Often, Always), and a total of 5 individual questions are reverse-coded. The CEBQ covers eight constructs of eating style (food responsiveness, emotional overeating, enjoyment of food, desire to drink, satiety responsiveness, slowness in eating, emotional undereating, and food fussiness). Constructs are organized in two dimensions, each consisting of 4 subscales. The Food Approach dimension consists of food responsiveness, emotional overeating, enjoyment of food, desire to drink; and the Food Avoidance dimension consists of satiety responsiveness, slowness in eating, emotional undereating, and food fussiness. The eight constructs are scored; the CEBQ does not have a summed score.

Child Nutrition Risk: The Nutrition Screening Tool for Every Preschooler (NutriSTEP^®^) assesses child nutrition risk [35]. NutriSTEP^®^ addresses 5 components of risk (food and nutrient intakes, physical growth, developmental and physical capabilities, other factors affecting food intake and eating behaviors, and physical activity and sedentary behavior) across 17 survey items. Response options for each item have an assigned value that is summed and categorized. Overall scores may range from 0 to 68; scores ≤20 indicate no nutrition risk, scores of 21−25 indicate moderate nutrition risk, and scores ≥26 indicate high nutrition risk.

Eating Competence: Parent/caregiver eating competence (EC), characterized as being flexible, comfortable, and positive with food and eating, and reliable about getting enough nourishing and enjoyable food to eat [36], was measured with the 16-item validated and reliability-tested Satter Eating Competence Inventory (ecSI2.0™). Advance permission from ecSI2.0™ developers was obtained to use the survey [37]. The ecSI2.0™ includes 4 subscales aligned with EC constructs: eating attitudes, food acceptance, internal regulation, and contextual skills. Each of the 16 items have 5 response options, assigned values ranging from 3 to 0, with possible scores ranging from 0 to 48, with higher numbers indicating greater EC. Scores ≥ 32 indicate EC [38,39].

Food Security: Household food security was assessed with the six-item short form of the U.S. Department of Agriculture food security survey module, which has been validated to identify food-insecure households and households with very low food security [40]. The questionnaire references food eaten in the household in the last 12 months and whether the respondent was able to afford the food that they needed. Each item has 3–4 response choices, and all items include an option of “Don’t Know”. Responses are summed with a possible range of 0–6; answers that affirm food insecurity score higher values. Participants are categorized in 1 of 3 groups: high or marginal food security among adults (score 0–1), low food security among adults (score 2–4), and very low food security among adults (score 5–6).

Demographics: Parent/caregiver demographics were self-reported and included respondent age, race, Hispanic/Latino ethnicity, education, gender, current weight and height (or pre-pregnancy weight for respondents who were currently pregnant or had recently given birth), and number of children in the household. Household participation in the past year for Supplemental Nutrition Assistance Program (SNAP), Supplemental Nutrition Assistance for Women, Infants and Children (WIC), and other income or food assistance programs, as well as current receipt of child care vouchers was queried.

Child Weight and Height: After parents/caregivers completed the survey, children were weighed without shoes and in light clothing using a calibrated, digital scale (SECA, Model 703 1321998), and weight was recorded to the nearest tenth of a kilogram. Height was measured to the nearest tenth of a centimeter, without shoes using a portable measuring rod (SECA Model 213). Both weight and height were measured in duplicate, and the two values were averaged.

### 2.3. Data Analysis

Children’s BMI-for-age z-scores were calculated using the Centers for Disease Control SAS Program [41] with SAS version 9.4. Scales were scored and summed according to survey directions. NutriSTEP^®^ low-risk category was compared to a group formed by combining moderate- and high-risk categories. To address small sample sizes of some options, individuals identifying their race as other than White (non-White) were collapsed into one category to compare with White participants; participants with <4-year college degree were collapsed to compare with those having a 4-year college degree or higher. Normality was assessed by Q-Q plots and skewness and kurtosis; absolute values ≤1 were accepted as normally distributed. Associations between sDOR.2-6y™ and CEBQ scales, NutriSTEP^®^, food security, and BMI-for-age z-scores were tested by Pearson r and Chi Square (or Fisher–Freeman–Halton Exact Test) for continuous (e.g., BMI-for-age z-scores, sDOR.2-6y™, NutriSTEP^®^ scores) and categorical variables (e.g., sDOR adherence/non-adherence or no nutrition risk/high or low nutrition risk), respectively. Mean differences between groups (e.g., sDOR.2-6y™ < 26 vs. ≥26, college-educated vs. not college-educated) for continuous variables (e.g., sDOR.2-6y™, NutriSTEP^®^ scores) were compared with independent sample *t*-test, or if group sizes were uneven, Mann–Whitney U. Block Dietary Data Systems Kids Food Screener results were provided by NutritionQuest, Berkeley, CA, USA [42]; records with <500 Kcal/day were excluded. Dietary component comparisons were made using Spearman’s rho correlation coefficient to address limited dietary sample size. Linear regression was used to predict NutriSTEP® scores from sDOR.2-6y™ score. Data were analyzed with SPSS version 28. 

This was an exploratory study of the sDOR.2-6y™. Therefore, despite multiple statistical tests, significance was set at *p* < 0.05. However, the increased potential for Type I errors was recognized by interpreting significant results with caution and reporting *p* values after adjusting with Holm’s correction for multiple comparisons [43]. This correction method addresses concerns with increased Type I errors, but with lower probability of making Type II errors, and thereby increasing statistical power. 

## 3. Results

### 3.1. Description of Participants

The parent/caregiver sample completing the original survey set from 27 August 2019 to 21 November 2019 (n = 91) were mostly White, highly educated, female (91%), food secure, and eating-competent, with children mostly three or four years of age; the mean age in 2019 was 3.5 ± 0.6 y. The response rate was ~32.5% (n = 91 responses of the approximately 280 accessible population). Parents’ ages ranged from 22 to 50 y, with a mean of 35.9 ± 5.1 y for those completing the 2019 survey (n = 91) and 35.8 ± 5.2 for those completing the 2021 items (n = 69). Children were identified as moderate or high nutrition risk for 23% of the sample completing the NutriSTEP^®^, which is comparable to other samples [18,44]. Parents’ self-reported heights and weights revealed similar proportions of normal weight and overweight/obesity, with a lower prevalence of child obesity from the measured heights and weights compared to nationally representative data [45]. These characteristics also described the subsample completing the approved version of the sDOR.2-6y™ from 8 June 2021 to 21 June 2021 (n = 69). In addition, the CEBQ, USDA Food Security Screener, and the ecSI2.0™ responses of the 2021 subsample were comparable to those only completing the 2019 survey. The sociodemographic, behavioral, and dietary survey responses were comparable between the 22 completing only the 2019 survey and the 69 providing responses in both 2019 and 2021. Details are provided in Table 1.

Within-participant response consistency was evident. For example, the NutriSTEP^®^ item, I have difficulty buying food to feed my child, was correlated with the USDA Food Security Screener Score (r = 0.60, *p* < 0.001, n = 87) and the frequency of worrying about money for food (r = 0.47, *p* < 0.001, n = 91). Additionally, the NutriSTEP^®^ item, I let my child decide how much to eat, was correlated with the sDOR.2-6y child autonomy domain (r = 0.25, *p* = 0.042, n = 69). The responses to the USDA Food Security Screener indicated more food insecurity (28% vs. 3%, *p* < 0.001) among those without a college degree (n = 18) than in those with a 4-year college degree or higher (n = 69).

The Cronbach α for ecSI 2.0 was 0.85. More than half of the parents were eating-competent (i.e., ecSI2.0 score of ≥32). The parent/caregiver eating competence was related to the child being at a lower nutrition risk (r = 0.32, *p* = 0.008, n = 69). Contextual skills were greater with a lower child nutrition risk (r = 0.49, *p* < 0.001), including the food and nutrition (r = 0.39, *p* = 0.001), physical activity (r = 0.44, *p* < 0.001), developmental capability (r = 0.29, *p* = 0.02), and other factors (r = 0.34, *p* = 0.004) components. Only *p* values ≤ 0.001 remained significant after applying the Holm correction factor. The NutriSTEP^®^ scores were significantly higher (indicating nutrition risk) for non-EC (n = 25) than EC parents (n = 44) (18.4 ± 6.1 vs. 15.2 ± 6.2; *p* = 0.038), but not when adjusted for multiple comparisons. Contextual skills were significantly higher (*p* = 0.008; adjusted *p* = 0.04) for parents with youth at a low nutrition risk (11.1 ± 2.1, n = 53) than at a moderate (9.2 ± 2.7, n = 11) or high risk (8.8 ± 2.8, n = 5). The EC status was not associated with self-reported parental BMI, education level, or race, with the exception of food acceptance being higher in non-White (n = 14) than White (n = 77) parents (7.0 ± 1.7 vs. 5.5 ± 2.2; *p* = 0.014; adjusted *p* = 0.07).

Parents’ food security (n = 91) was associated with eating competence, with food-insecure parents having lower eating competence (r = −0.25, *p* = 0.016), internal regulation (r = −0.24, *p* = 0.02), and contextual skills (r = −0.40, *p* < 0.001). These relationships remained significant (contextual skills) or a trend when adjusted for multiple comparisons. Parents with high or marginal food security (n = 84) had greater contextual skills than those with low or very low food security (n = 7) (10.8 ± 2.3 vs. 7.6 ± 4.2; *p* = 0.002; adjusted *p* = 0.01). Among those completing both survey opportunities, parents with high or marginal food security (n = 66) had greater contextual skills than parents with low or very low food security (n = 3) (10.8 ± 2.3 vs. 7.3 1.2; *p* = 0.013; adjusted *p* = 0.065). Eating competence was higher in parents whose CEBQ responses showed child food enjoyment (r = 0.21, *p* = 0.042; adjusted *p* =0.168) with slower eating at mealtime (r = 0.26, *p* = 0.01; adjusted *p* = 0.05).

### 3.2. Adherence to Satter Division of Responsibility (sDOR.2-6y™)

The sDOR.2-6y™ scores (n = 69) ranged from 13 to 34 out of a possible 0–68. Table 1 includes the means and standard deviations for the overall scale and the two domains, as defined by Lohse and Mitchell [18]. The survey mean of 26.1 ± 3.9 was comparable with the mean reported by Lohse and Mitchell of 25.9 ± 3.3. Cronbach α = 0.57, which is greater than the 0.32 previously reported [18].

Although the entire survey was not administered on two occasions, test–retest reliability can be suggested by comparing the findings from the nine items completed on both occasions. The nine items summed up to 19.4 ± 3.3 in 2019 and 20.2 ± 3.0 in 2021. These were significantly correlated (r = 0.61, *p* < 0.001, n = 69) and were not significantly different with a paired *t*-test.

### 3.3. sDOR.2-6y™ and Child BMI-for-Age

Among parents/caregivers completing both surveys, the child BMI-for-age z-scores were higher (0.91 ± 1.15) for parents without a college degree (n = 12) than for parents with a college degree (n = 53; *p*.05 ± 0.85; *p* = 0.004), but the child means for both groups were within the healthy range as per the Centers for Disease Control guidelines [46]. sDOR.2-6y™ was not related to parents’ BMI (from self-reported height and weight; r = 0.22, *p* = 0.08, n = 65) or measured child BMI-for-age z-score (r = 0.11, *p* = 0.39, n = 69). sDOR.2-6y™ scores were not significantly different between children with BMI-for-age z-scores designated as overweight/obese (n = 11; 26.8 ± 3.3) or not (n = 58; 25.9 ± 4.0). Additionally, no sDOR.2-6y™ domain score differences between children with healthy or overweight/obese BMI-for-age z-scores were statistically or practically significant.

### 3.4. sDOR.2-6y™ and Parent Perceptions of Child Eating Behavior

The CEBQ Cronbach α was 0.84. Findings, unadjusted for multiple comparisons, showed that adherence to sDOR or its components was associated with three of the eight CEBQ constructs, including greater food enjoyment, a lower desire to drink, and lower satiety responsiveness. When adjusted for multiple comparisons, a lower desire to drink continued to be associated with adherence to overall sDOR and the sDOR child autonomy domain (Table 2). For those completing the sDOR.2-6yTM, the CEBQ slowness in eating and emotional overeating subscales were correlated with the child BMI-for-age z-scores (n = 69, r = −0.35, *p* = 0.003; r = 0.32, *p* = 0.008 respectively). Only the CEBQ emotional overeating subscale score differed between overweight/obese (n = 11) and healthy weight (n = 52) youth (9.5 ± 2.3 vs. 7.6 ± 2.6, *p* = 0.34). For the larger sample completing only the first survey (n = 91), BMI-for-age z-scores were significantly associated with slowness in eating (r = −0.24, *p* = 0.021), emotional overeating (r = 0.23, *p* = 0.026), and a desire to drink (r = 0.22, *p* = 0.37). The CEBQ subscale scores did not differ between overweight or obese and healthy weight youth.

Parents with lower adherence to sDOR, defined as <26, reported significantly more child food fussiness, desire to drink, and less enjoyment of food (Table 3).

### 3.5. sDOR.2-6y and Household Food Security

The Cronbach α of the USDA food security screener was 0.80. The associations of sDOR.2-6y™ scores with the USDA food security findings were limited, because only three adult households were considered food insecure. The sDOR.2-6y™ scores did not differ between food-secure and -insecure parents when controlling for either White vss non-White or 4-year college degree status.

### 3.6. sDOR.2-6y and Nutrition Risk

The NutriSTEP^®^ Cronbach α was 0.63. Adherence to sDOR was correlated with a lower nutrition risk (NutriSTEP^®^ score) (r = 0.26, *p* = 0.03) but not when adjusted for multiple comparisons (Table 2). Using the nutrition risk findings from the validated NutriSTEP^®^ scale to indicate True Negative and True Positive indicators, the sDOR.2-6y™ sensitivity and specificity were 0.63 and 0.64 with a nutrition risk cutoff of <26. Using the sDOR.2-6y™ nutrition risk cutoff <26 labeled 29 (42%) children as being at a nutritional risk. Child BMI-for-age z-scores were not significantly different between those with sDOR.2-6y™ scores <26, even when controlling for being non-White or not having a college degree. The NutriSTEP^®^ score could be predicted from the sDOR.2-6y™ with the equation 27.364 − (0.422 × sDOR.2-6yTM score); *p* = 0.03 95% CI −0.80 to −0.04.

### 3.7. sDOR.2-6y™ and Eating Competence

In this sample, sDOR.2-6y TM and eating competence were not related.

### 3.8. sDOR.2-6y™ and Dietary Intake

An eligible dietary screen was submitted by 86 participants. The dietary intake is shown in Table 4. A total of 65 participants had an eligible NutriSTEP^®^ score and dietary intake. The unadjusted total NutriSTEP^®^ score was inversely correlated with the vegetable (not including potatoes or legumes), dairy, and total protein dietary components of the Block Kids Food Screener, indicating a lower nutrition risk with a higher intake of these foods/nutrients. The food and nutrient subscale score showed similar unadjusted relationships with the intake of vegetables (not including potatoes or legumes), dairy, legumes, fiber components, and total protein.

The relationships with vegetables (not including potatoes or legumes) and dairy with total the NutriSTEP^®^ score continued to be significant when adjusted for multiple comparisons (Supplementary Table S1). A higher intake of vegetables (not including potatoes or legumes), dairy, fiber, and protein was noted for children at a low nutrition risk (n = 50) compared to those at a moderate or high nutrition risk (n = 15) However, these relationships were not significant when adjusted for multiple comparisons (Supplementary Table S2).

A lower intake of potatoes and French fries among children with caregivers who have greater sDOR adherence to child autonomy in feeding was noted (overall child autonomy domain rho= −0.28; n = 65; *p* = 0.03; adjusted *p* = 0.72). The sDOR.2-6y™ scores of the dietary participants were nearly identical to the total sample (e.g., total sDOR.2-6y™ score 26.2 ± 4.0) vs. 26.1 ± 3.9; parent leadership domain 16.1 ± 2.6 vs. 16.1 ± 2.6, and child autonomy domain 10.1 ± 2.0 vs. 10.0 ± 2.0, respectively).

## 4. Discussion

This study was the first to examine the potential differences in parent adherence to sDOR by child BMI-for-age and food security status and to examine the relationship between sDOR2.6y™ and CEBQ. Our results indicate that parental sDOR adherence is not correlated with the child’s BMI-for-age or food security status. The sDOR.2-6y™ demonstrated construct and convergent criterion validation in the ability of sDOR.2-6y™ to identify young children at nutrition risk, as identified by the NutriSTEP^®^. Moreover, this study identified correlations between the sDOR2.6y™ and three CEBQ constructs, but a limited association between sDOR2.6y™ and dietary intake was noted.

Child obesity is a complex issue with environmental, genetic, societal, political, and psychological aspects [47]. Several studies support links among the way that parents feed their children (i.e., parental feeding behaviors), child eating behaviors, and child weight status [24,25,26,48]. A cross-sectional study of more than 3000 4-year-olds aligned parent restriction and lower satiety responsiveness with higher overweight and obesity [25]. A review of 31 articles revealed higher child overweight and obesity with parental restrictive feeding and an indulgent feeding pattern [48], although the relationship is less clear in longitudinal studies [22]. In-depth semi-structured interviews with a sample of 71 parents of racially diverse children ages 2–5 years (approximately 50% with BMI-for-age ≥ 85th percentile) who participated in the Special Supplemental Nutrition Program for Women, Infants and Children (WIC) revealed support for the sDOR tenets among the caregiver feeding practices of children who were at a healthy BMI. Caregivers of children in the 5th-85th percentile for BMI-for-age were more likely to report having a consistent routine for providing meals, using a guided choices approach, serving small portions of food during mealtimes, and trusting their child’s hunger and satiety cues compared to caregivers of children ≥ 85th percentile for BMI-for-age [49].

In light of previous research, it is reasonable to hypothesize that greater parental adherence to the sDOR is associated with lower overweight and obesity in young children. However, results from the present study do not demonstrate a relationship between sDOR adherence and the BMI-for-age z-score or weight category. Moreover, no sDOR.2-6y™ domain score differences between children with healthy or overweight/obese BMI-for-age z-scores were statistically or practically significant. Ecologic models, such as Brofenbenner’s Social Ecologic Model [50], have been widely used in public health and obesity initiatives. Ecological models focus on changing personal behaviors while accounting for the influences of social, physical, and political environments. As such, there is a focus on policy and structural changes to produce new behavior, while maintaining an individual’s agency. The sDOR.2-6y™ only measures the parents’ feeding behavior (an individual-level characteristic) and does not account for the influences of other systems on individual behavior. Given what is already known about the complexities of childhood obesity, it is not surprising that sDOR adherence alone is not related to child BMI-for-age.

This study was the first to compare sDOR.2-6y™, a measure of parents’ feeding behaviors, with the CEBQ, which measures parents’ perceptions of the child’s eating behavior. Early childhood is a particularly important developmental period to study the relationship between parent feeding and child eating behaviors, given that children are still highly dependent on caregivers but are in a period of seeking more autonomy. After adjustment for multiple comparisons, only the CEBQ subscale desire to drink was correlated with sDOR.2-6y™ and the child autonomy domain. In the sample of n = 69, sDOR.2-6y™ was not associated with BMI-for-age, but the CEBQ subscales of slowness in eating and emotional overeating were correlated with the BMI-for-age z-score, and the emotional overeating subscale differed between children categorized as being a healthy weight versus overweight/obese. In the larger sample of n = 91, lower slowness in eating and greater emotional overeating and desire to drink were correlated with the BMI-for-age, but no differences by BMI-for-age category were noted. Despite the fact that the CEBQ has been presented as a tool to identify child eating behaviors associated with obesogenic behaviors [51], the present results do not support a robust relationship between child eating behavior constructs as measured by CEBQ and whether or not the child’s BMI is classified as healthy or overweight/obese. Several considerations can be examined in light of this difference. Firstly, the validation study of the CEBQ [14] determined an association between CEBQ and eating behaviors linked with obesity, but the study did not measure the child’s weight (either self-reported or investigator-measured). Findings by Ek et al. [52] demonstrated a positive correlation between the child BMI Standard Deviation Score (BMI SDS) and the Food Approach subscale of CEBQ (r = 0.579, *p* < 0.001) and a negative correlation with the Food Avoidance subscale CEBQ (r = −0.396, *p* < 0.01). However, BMI was only examined as the continuous BMI SDS. Differences in the mean scores of food approach and food avoidance by BMI category (i.e., underweight, healthy weight, overweight, and obese) were not examined. Thus, it can be difficult to assess the clinical and practical significance of these findings, particularly with a variable such as child BMI-for-age, where anything from the 5th–85th percentile is categorized as “healthy”. Clinical practice supports “healthy” growth at a continuum of BMI-for-age percentiles when growth tracks along a percentile and avoids pathologizing tracking along percentiles that are higher, but still within the 5th–85th percentile range. In addition, the Ek et al. sample was Swedish, but the CEBQ was developed in the United Kingdom and validated using confirmatory factor analysis (CFA) with a United States sample [53]. It is feasible that cultural differences may influence the construct validation. Similar to the present findings, Gregory et al. [54] found that the child BMI z-score was not an independent predictor of the CEBQ parents’ report of child eating behavior (either Food Approach or Food Avoidance). However, mothers who reported a higher level of concern about their child being underweight reported answers that scored their child higher on the Food Avoidance subscale, and mothers who reported a higher level of concern about their child being overweight reported answers that scored their child higher on the Food Approach subscale. This suggests that parents may respond to issues with the child’s perceived weight or weight concerns, rather than the child’s actual weight. In the present study, the measured weights and heights in the sample show that in the original sample of n = 91 children, 69 (76%) were a healthy weight, 71 (8%) were underweight, 13 (14%) were overweight, and 2 (2%) were obese. Future research with a larger sample might yield different results.

Household food insecurity, defined as household-level economic or social conditions of limited or uncertain access to food [55], affects roughly 12.5% of U.S. households with children [56]. Limited research exists to examine parent feeding practices by food security status. Trofholz and colleagues examined family meal characteristics and parent feeding practices among parents of children ages 5–7 years by household food security using both quantitative and qualitative methods [57]. Interestingly, the quantitative results showed few differences in parent feeding practices by food security status, but the qualitative data indicated several differences in parent feeding practices by food security status and related to the sDOR model. For example, food-secure families qualitatively reported a greater frequency of requiring their child to try all the foods served at a family meal compared to food-insecure families, but food-secure families were less likely to require children to eat all their food /clean their plate compared to food-insecure families. Food-secure families were more likely to report offering children an alternate meal if the child did not like the food, whereas this behavior was rarely mentioned by food-insecure families. Food-insecure parents were more likely to report that their role at the family meal was to make sure that their child ate compared to food-secure parents, and food-insecure parents were more likely to restrict their child’s intake if they felt that the child was eating too much compared to food-secure parents. The authors suggest that a possible explanation for the discrepancy between the quantitative and qualitative results could be that the quantitative questions (e.g., “Did you encourage your child to eat more at this meal?”) did not resonate/make sense with families in the same way that they were able to explain themselves in the qualitative interview (e.g., parents require children to try foods at family meals but do not see this as an encouragement to eat more). A benefit of the sDOR.2-6y™ is the validation of the parent’s response with the video capture of mealtime in the family home, which ensures that the parent’s survey response aligns with actual behavior [17]. The sDOR.2-6y™ video-captured validation sample was largely low-income, but the food security status was not measured. Although the present study did not find any differences in caregiver adherence to sDOR by food security status, the number of food-insecure households was relatively small. Further investigation of parental qualitative and quantitative responses to sDOR tenets by food security status is an important topic for future research.

Convergent criterion validation was demonstrated in the ability of sDOR.2-6y™ to identify young children at nutrition risk, as indicated by the NutriSTEP^®^. A 2021 investigation determined that parents of children at a high or medium nutrition risk as determined by NutriSTEP^®^ had lower sDOR.2−6y™ scores compared with children at a low nutrition risk (*p* = 0.004) [14]. Moreover, each 1-point increase in sDOR.2-6y™ decreased the odds of being classified as moderate or high nutrition risk by 21% (95% confidence interval, 0.675–0.918; *p* = 0.002). These findings align with results from the present study demonstrating a statistically significant association between the sDOR.2-6y™ score and NutriSTEP^®^ score using simple linear regression (*p* = 0.03) and a significant correlation between the sDOR.2-6y™ and NutriSTEP^®^ (r = −0.26, *p* = 0.03), although the correlation was not significant after adjustment for multiple comparisons.

No previous research has investigated children’s dietary intake in relation to parental sDOR adherence. The present study found that a greater sDOR adherence to child autonomy was associated with a lower intake of potatoes and French fries among children, but the relationship was not significant after adjustment for multiple comparisons. There is a strong body of research showing that dietary quality is higher among families that eat together, which is a component of the sDOR model [58], and continued investigation of dietary intake and dietary quality by sDOR adherence is warranted, particularly using more robust methods of dietary assessment. The present study utilized the Block Kids Food Screener, which is limited in the number of foods/beverages that are ascertained and is not able to capture measures of dietary quality such as the Healthy Eating Index [59]. Another consideration is that all children attended an early childhood education center, the majority of which were full-time child care settings. Thus, most children were consuming one or more meals plus snack(s) away from their parent/caregiver. Therefore, this sample of parents/caregivers may have encountered challenges to accurately recall what their child ate in the previous week (the time frame of the Block Kids Food Screener).

The present study has several strengths, including investigator-measured heights and weights of children, the measurement of household food security, and a comparison of the sDOR.2-6y™ with the previously validated CEBQ and NutriSTEP^®^. Limitations of the study include a relatively small sample size with limited racial and educational diversity and few households experiencing food insecurity. Participants were offered a USD 30 gift card to compensate for the time and effort of completing the initial survey. It is possible that the provision of this incentive biased participation in ways that we are unable to measure. Although the sample size of the present study was smaller than a previous validation study sample, the standard deviation and range were similar and suggest that the variability of the sample was adequate. Potential avenues for future exploration include a sample with greater racial, economic, educational, and geographic diversity. In addition, it would be useful to perform criterion validation with measures of children’s quality of life and ingestive behaviors, especially with children who have special needs with dietary impact potential, e.g., diabetes or allergies.

## 5. Conclusions

This study affirmed that the sDOR.2-6y^TM^ is a useful screening tool to identify child nutrition risk. In this sample of children attending an early childhood education center, sDOR.2-6y™ was not associated with the child’s BMI-for-age, household food security status, or dietary intake. Incorporating the sDOR.2-6y^TM^ into routine pediatric and WIC assessments could prove to be a cost-efficient, useful preventive and public health measure. In addition, our findings support efforts to educate parents about sDOR and adopt an eating-competent approach to food.

## Figures and Tables

**Table 1:** Demographic and behavioral characteristics of participating parents of preschool-age children ^1^.

	**Original Sample of Parents and Children ^2^**	**Second Survey Administration Sample of Parents and Children ^3^**
**Child’s Age (y)** 3 4 5	52 (57) 36 (40) 3 (3)	35 (51) 32 (46) 2 (3)
**Race** Hispanic American Indian/Alaskan Native Asian Black White	4 (4) 2 (2) 5 (6) 6 (7) 77 (86)	3 (4) 0 (0) 5 (7) 5 (7) 58 (85)
**Highest Education** High School/GED Some College/Training 4-year college degree Post-graduate college	1 (1) 17 (20) 28 (42) 41 (47)	1 (2) 11 (17) 22 (34) 31 (48)
**Worry about having enough money for food?** Never Rarely Sometimes Often Always	49 (56) 24 (28) 11 (13) 2 (2) 1 (1)	38 (58) 18 (28) 7 (11) 2 (3) 0 (0)
**Assistance Programs** SNAP WIC TANF Medical Assistance Benefits Medicaid LiHEAP Food Bank Childcare Vouchers	7 (8) 6 (7) 1 (1) 3 (3) 7 (8) 2 (2) 1 (1) 4 (4)	5 (8) 5 (8) 1 (1) 2 (3) 6 (9) 2 (3) 1 (1) 2 (3)
**Parent BMI** Mean ± SD Range Underweight Normal weight Overweight Obese	27.5 ± 7.39 18.34–54.70 3 (3) 36 (41) 26 (30) 22 (25)	27.9 ± 8.24 18.34–54.70 3 (5) 28 (43) 16 (25) 18 (28)
**Child BMI-for-age z-score** Mean ± SD Range Moderate Malnutrition Mild Malnutrition Normal Weight Overweight Obese Severely Obese	0.25 ± 0.88 −2.22 to 3.63 1 (1) 6 (7) 69 (76) 13 (14) 1 (1) 1 (1)	0.21 ± 0.94 −2.22 to 3.63 1 (1) 5 (7) 52 (75) 9 (13) 1 (1) 1 (1)
**ecSI 2.0^TM 4^** Total Score, Mean ± SD Range Median Subscales, Mean ± SD Eating attitudes Food acceptance Internal regulation Contextual skills Not EC EC	34.2 ± 6.3 17–48 33.5 13.7 ± 2.3 5.7 ± 2.2 4.3 ± 1.1 10.5 ± 2.6 33 (36) 58 (64)	34.2 ± 5.9 24–48 33.5 13.6 ± 2.3 5.7 ± 2.2 4.3 ± 1.0 10.7 ± 2.4 25 (36) 44 (64)
**CEBQ ^5^, Mean**±**SD** Food Fussiness Enjoyment of Food Satiety Responsiveness Slowness in Eating Emotional Undereating Emotional Overeating Food Responsiveness Desire to Drink	18.9 ± 4.7 15.0 ± 2.2 15.6 ± 2.5 12.0 ± 2.9 12.0 ± 3.2 7.6 ± 2.6 12.6 ± 3.6 9.3 ± 3.0	18.6 ± 4.6 15.2 ± 2.3 15.4 ± 2.5 12.1 ± 2.7 11.9 ± 3.3 7.9 ± 2.6 12.8 ± 3.6 9.3 ± 3.0
**USDA Food Security Score ^6^, Mean**±**SD** High or Marginal Food Security Low Food Security Very Low Food Security	0.5 ± 1.2 84 (92) 4 (4) 3 (3)	0.4 ± 1.1 66 (96) 1 (1) 2 (3)
**sDOR.2-6y^TM 7,8^, Mean**±**SD** Total Score Range Median Parent Leadership with Feeding Child Autonomy About Eating		26.1 ± 3.9 13–34 26 16.1 ± 2.6 10.0 ± 2.0
**NutriSTEP^® 8,9^ Components, Mean**±**SD** Total Score Food and Nutrient Intakes Physical Growth Developmental and Physical Capabilities Other factors affecting food intake and eating behaviors Physical activity and sedentary behavior Risk Categories, n (%); Mean ± SD Low Risk Moderate Risk High Risk		16.4 ± 6.3 11.4 ± 3.8 0.5 ± 1.7 0.2 ± 0.4 3.6 ± 2.1 1.2 ± 1.8 53 (77); 13.7 ± 3.8 11 (16); 22.9 ± 1.4 5 (7); 30.6 ± 4.0

^1^ Table entries are n (%) unless otherwise noted. ^2^ n varies from 87 to 91. ^3^ n varies from 65 to 69. ^4^ ecSI 2.0^TM^ possible score 0–48; possible subscale scores: eating attitudes, 0–18; food acceptance, 0–9; internal regulation, 0–6; contextual skills, 0–15. ^5^ CEBQ possible subscale scores: food fussiness, 9–30; enjoyment of food, 9–20; satiety responsiveness, 10–22; slowness in eating, 6–20; emotional undereating, 4–19; emotional overeating, 4–15; food responsiveness, 5–22; desire to drink, 3–15. ^6^ USDA food security possible score 0–6; high or marginal food security, 0–1; low food security, 2–4; very low food security, 5–6. ^7^ sDOR.2-6y^TM^ measures adherence to the Satter Division of Responsibility in Feeding. Total overall score: 13–34. Possible scores: parent leadership with feeding, 0–21; child autonomy in eating, 0–15. ^8^ Scored values not available for original sample. ^9^ NutriStep^®^ consists of 17 items with possible scores of 0–68. Range for this sample was 6–37. Risk categories: Low ≤ 20; Moderate 21–25; High ≥ 26. Possible component scores: food and nutrient intakes, 4–21; physical growth and physical activity and sedentary behavior, 0–8; developmental and physical capabilities, 0–2; other factors affecting food intake and eating behaviors, 0–11. Abbreviations: ecSI 2.0^TM^, Satter eating competence inventory; EC, eating-competent; CEBQ, Child Behavior Eating Questionnaire; sDOR, Satter Division of Responsibility in Feeding.

**Table 2:** Selected comparisons of sDOR.2-6y^TM 1^ and subscale scores with NutriSTEP^® 2^ and component scores and Child Eating Behavior Questionnaire ^3^ scales in parents of preschool-age children (n = 69).

Score 1	Score 2	r ^4^	*p* Unadjusted	*p* Adjusted ^5^
sDOR.2-6y^TM^	NutriSTEP^®^	−0.26	0.030	0.068
sDOR.2-6y^TM^	NutriSTEP^®^ Other Factors	−0.40	<0.001	<0.018
sDOR.2-6y^TM^ Child Autonomy	NutriSTEP^®^	−0.26	0.035	0.068
sDOR.2-6y^TM^ Child Autonomy	NutriSTEP^®^ Other Factors	−0.31	0.004	0.068
sDOR.2-6y^TM^ Parent Leadership with Feeding	NutriSTEP^®^ Other Factors	−0.34	0.004	0.068
sDOR.2-6y^TM^	CEBQ Enjoyment of Food	0.25	0.036	0.108
sDOR.2-6y^TM^	CEBQ Desire to Drink	−0.34	0.003	0.006
sDOR.2-6y^TM^ Child Autonomy	CEBQ Satiety Responsiveness	−0.25	0.040	0.12
sDOR.2-6y^TM^ Child Autonomy	CEBQ Desire to Drink	−0.47	<0.001	<0.003
sDOR.2-6y^TM^ Parent Leadership with Feeding	CEBQ Enjoyment of Food	0.25	0.036	0.108

^1^ sDOR.2-6y^TM^ measures adherence to the Satter Division of Responsibility in Feeding. Possible scores: parent leadership with feeding, 0–21; child autonomy about eating, 0–15. ^2^ NutriStep^®^ consists of 17 items with possible scores of 0–68. Risk categories: Low ≤ 20; Moderate 21–25; High ≥ 26. Possible component scores: food and nutrient intakes, 0–21; physical growth and physical activity and sedentary behavior, 0–8; developmental and physical capabilities, 0–2; other factors affecting food intake and eating behaviors, 0–11. ^3^ CEBQ possible subscale scores: food fussiness, 9–30; enjoyment of food, 9–20; satiety responsiveness, 10–22; slowness in eating, 6–20; emotional undereating, 4–19; emotional overeating, 4–15; food responsiveness, 5–22; desire to drink, 3–15. ^4^ Pearson r correlation coefficient. ^5^ Holm Correction for multiple comparisons applied. sDOR.2-6y^TM^ and NutriStep^®^—18 total tests; sDOR.2-6y^TM^ and each CEBQ scale—3 total tests. Abbreviations: CEBQ, Child Behavior Eating Questionnaire; sDOR, Satter Division of Responsibility in Feeding.

**Table 3:** Comparison of child eating behavior scale scores between categories of nutrition risk, as defined by sDOR.2-6y^TM^ scores using independent *t*-test.

<26 sDOR.2-6y^TM^ Indicates Nutrition Risk			
CEBQ ^1^ Scale	At Risk (n = 29)	Not at Risk (n = 40)	*p* Value
Food Fussiness	20.3 ± 4.4	17.4 ± 4.3	0.007
Enjoyment of Food	14.4 ± 2.4	15.9 ± 2.1	0.008
Desire to Drink	10.4 ± 2.7	8.5 ± 3.1	0.01
Satiety Responsiveness	15.8 ± 2.6	15.1 ± 2.4	0.21
Slowness in Eating	12.2 ± 2.6	11.9 ± 2.8	0.64
Emotional Undereating	12.6 ± 2.4	11.4 ± 3.8	0.13
Emotional Overeating	8.5 ± 2.3	7.4 ± 2.8	0.09
Food Responsiveness	13.4 ± 3.5	12.3 ± 3.6	0.020

^1^ CEBQ possible subscale scores: food fussiness, 9–30; enjoyment of food, 9–20; satiety responsiveness, 10–22; slowness in eating, 6–20; emotional undereating, 4–19; emotional overeating, 4–15; food responsiveness, 5–22; desire to drink, 3–15. Abbreviations: CEBQ, Child Behavior Eating Questionnaire; sDOR, Satter Division of Responsibility in Feeding.

**Table 4:** Average daily dietary intake of preschool youth (n = 86) collected from the Block Dietary Data Systems Kids Screener ^1^.

Dietary Feature	Mean ± SD	Range
Fruit/Fruit Juice (CE) ^2^	1.4 ± 0.7	0.02–4.3
Vegetables (not potatoes/legumes) (CE)	0.5 ± 0.3	0.07–1.3
Potatoes (including French fries) (CE)	0.1 ± 0.1	0.00–0.6
Whole Grains (ounce-equivalent)	0.4 ± 0.3	0.00–1.3
Meat, Poultry, Fish (ounce-equivalent)	1.5 ± 0.8	0.2–4.6
Dairy (CE)	1.7 ± 0.8	0.1–3.7
Legumes (CE)	0.03 ± 0.06	0.00–0.4
Added Sugars/Syrups (teaspoons)	3.8 ± 1.9	0.5–10.6
Kcals	916.0 ± 246.5	501.3–1924.7
Protein (g)	40.8 ± 12.1	22.2–81.2
Fat (g)	36.1 ± 11.1	16.8–65.9
Carbohydrate (g)	110.9 ± 12.5	55.6–268.1
Fiber (g)	8.5 ± 3.1	3.8–20.6

^1^ Sample excluded records with Kcal intake < 500 (n = 5). ^2^ Cup-equivalent (CE).

## Data Availability

The data presented in this study are available on request from the corresponding author, Elizabeth Ruder. The data are not publicly available due to due to containing information that could compromise the participants’ privacy.

## Supplementary Materials

The following supporting information can be downloaded at https://www.mdpi.com/article/10.3390/nu16060767/s1: Table S1: Selected NutriSTEP^®^ and Food and Nutrition Component scores (n = 65) compared with dietary intake components in preschoolers using Spearman Rho correlation coefficient; Table S2: Dietary intake of preschool children at low nutrition risk (n = 50) compared to the group with moderate (n = 10) or high nutrition risk (n = 5) as defined by NutriStep^®^ using Mann–Whitney U test.
