# Supplementary material for: The sDOR.2-6y™ Is a Valid Measure of Nutrition Risk Independent of BMI-for-Age z-Score and Household Food Security Status in Preschool Aged-Children
Source: Nutrients. 2024 Mar 7;16(6):767. doi: 10.3390/nu16060767 (PMC10974798; doi:10.3390/nu16060767)
Supplement: Supplementary file 1 [file nutrients-16-00767-s001.zip › nutrients-2877266-supplementary.pdf]

Supplementary Table S1. NutriSTEP® and Food and Nutrition Component scores (n=65) compared with dietary intake components in preschoolers using Spearman Rho correlation coefficient

	**NutriSTEP® Score[a]**		**NutriSTEP® Food & Nutrition Component[b]**	
**Selected Dietary Components[c]**	**rho**	***P* value**	**rho**	***P* value**
Fruit/Fruit Juice (CE)	-0.16	NS	-0.22	0.08
Vegetables (no potatoes/legumes) (CE)	-0.39	<0.001	-0.43	<0.001
Dairy (CE)	-0.38	0.002	-0.34	0.005
Whole Grain (ounce equivalents)	-0.22	0.08	-0.12	NS
Legumes (CE)	-0.24	0.06	-0.25	0.043
Fiber (g)	-0.24	0.06	-0.28	0.026
Total Protein (g)	-0.25	0.04	-0.29	0.019

[a]Possible score for NutriSTEP is 0 - 68

[b]Possible score for NutriSTEP Food and Nutrition Components is 0 - 36

[c]Dietary data from respondents reporting an average ≥ 500 Kcal/day using the Block Dietary Data Systems Kids Food Screener for three days.CE=Cup Equivalents

Supplementary Table S2. Dietary intake of preschool children at low nutrition risk (n=50) compared to the group with moderate (n=10) or high nutrition risk (n=5) as defined by NutriStep® using Mann-Whitney U test.

**Selected Dietary Components**[a]	**Low Nutrition Risk**[b]	**Moderate or High Nutrition Risk**[c]	***P* value**[d]
Fruit/Fruit Juice (CE)	35.24	25.53	0.08
Vegetables (no potatoes/legumes) (CE)	35.54	24.53	0.048
Dairy (CE)	36.40	21.67	0.008
Whole Grain (ounce equivalents)	35.29	25.37	0.08
Carbohydrate (g)	35.34	25.20	0.07
Fiber (g)	35.64	24.20	0.04
Total Protein (g)	35.53	24.57	0.049

[a]Dietary data from respondents reporting an average ≥ 500 Kcal/day using the Block Dietary Data Systems Kids Food Screener for three days; CE=Cup Equivalents

[b]Mean ranks from Mann-Whitney U for NutriSTEP® score < 21

[c]Mean ranks from Mann-Whitney for NutriSTEP® score 21 - 25 (moderate risk) ≥ 26 (high risk)

[d]Values from Mann-Whitney U test
